# Supplementary material for: Predicting visual function by interpreting a neuronal wiring diagram
Source: Nature. 2024 Oct 2;634(8032):113–23. doi: 10.1038/s41586-024-07953-5 (PMC11446822; doi:10.1038/s41586-024-07953-5)
Supplement: Supplementary file 6 — CRF and ERF predictions for individual TmY4 and TmY9 cells. Analogous to Supplementary Data 3, but for TmY target types. Shown are the top four monosynaptic pathways, the strongest pathway passing through each of the top ten intermediary types (ranking from Extended Data Fig. 7), and the trisynaptic pathway Tm1–TmY–Dm3–TmY (see the section entitled Prediction of spatial normalization). [file 41586_2024_7953_MOESM6_ESM.zip › DataS4/TmY9qΓèÑ/720575940629163779.pdf]

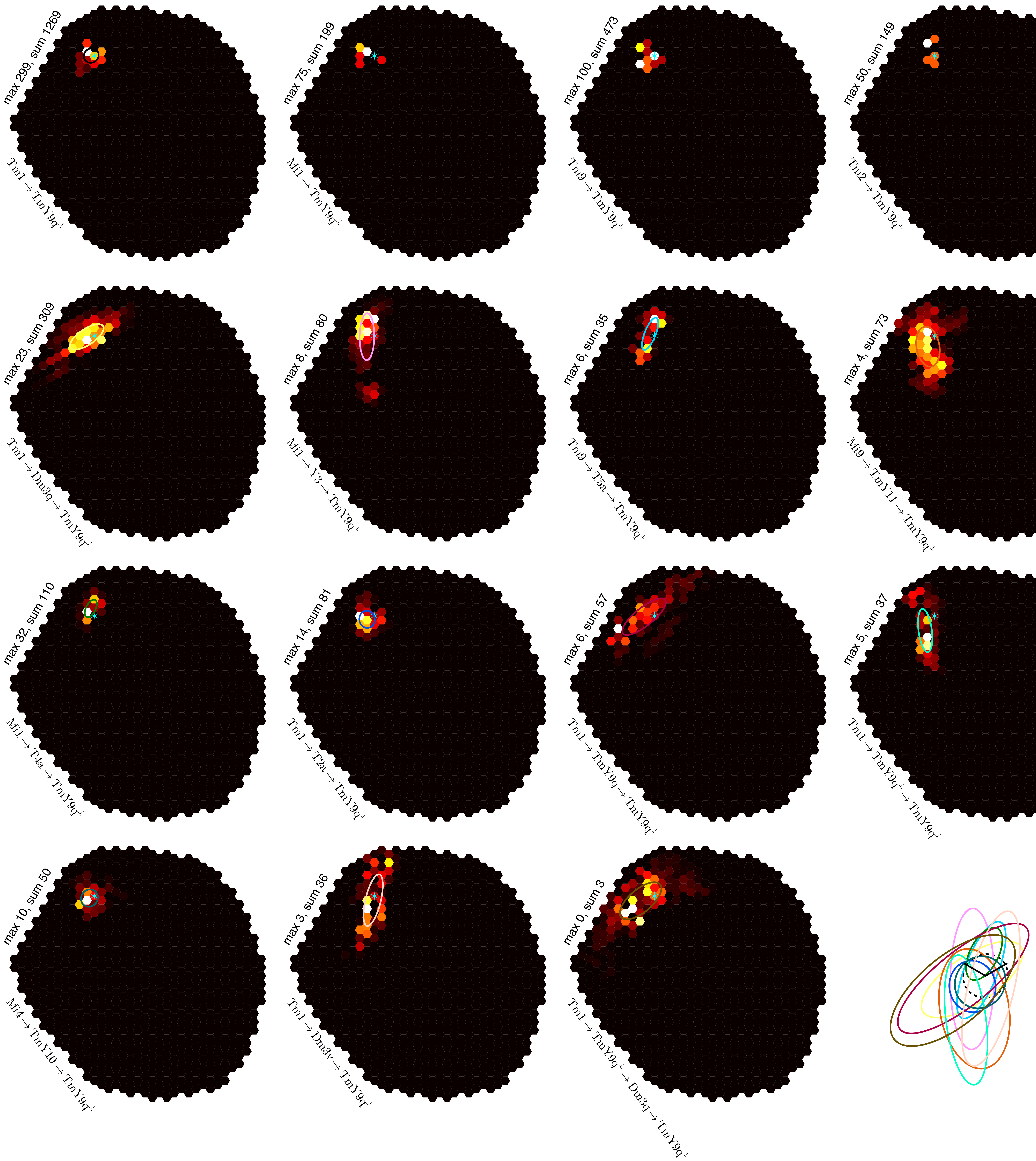

max 299, sum 1269
Tm1 → TmY9q⊥
max 75, sum 199
Mi1 → TmY9q⊥
max 100, sum 473
Tm9 → TmY9q⊥
max 50, sum 149
Tm2 → TmY9q⊥
max 23, sum 309
Tm1 → Dm3q → TmY9q⊥
max 8, sum 80
Mi1 → Y3 → TmY9q⊥
max 6, sum 35
Tm9 → T5a → TmY9q⊥
max 4, sum 73
Mi9 → TmY11 → TmY9q⊥
max 32, sum 110
Mi1 → T4a → TmY9q⊥
max 14, sum 81
Tm1 → T2a → TmY9q⊥
max 6, sum 57
Tm1 → TmY9q → TmY9q⊥
max 5, sum 37
Tm1 → TmY9q⊥ → TmY9q⊥
max 10, sum 50
Mi4 → TmY10 → TmY9q⊥
max 3, sum 36
Tm1 → Dm3v → TmY9q⊥
max 0, sum 3
Tm1 → TmY9q⊥ → Dm3q → TmY9q⊥
